# Service provision in the wake of a new funding model for community pharmacy

**DOI:** 10.1186/s12913-018-3120-z

**Published:** 2018-05-02

**Authors:** Alesha J. Smith, Shane L. Scahill, Jeff Harrison, Tilley Carroll, Natalie J. Medlicott

**Affiliations:** 1 School of Pharmacy University of Otago, PO Box 56, Dunedin, New Zealand; 2 School of Management Massey University, Auckland, New Zealand; 3 School of Pharmacy University of Auckland, Auckland, New Zealand

**Keywords:** New Zealand, Community pharmacy, Patient-centred services, New funding models, Service provision, Policy change

## Abstract

**Background:**

Recently, New Zealand has taken a system wide approach providing the biggest reform to New Zealand community pharmacy for 70 years with the aim of providing more clinically orientated patient centred services through a new funding model. The aim of this study was to understand the types of services offered in New Zealand community pharmacies since introduction of the new funding model, what the barriers are to providing these services.

**Method:**

A survey of all community pharmacies were undertaken between August, 2014 and February, 2015. Basic descriptive statistics were completed and group comparisons were made using the chi squared test with significance set at *p* < 0.05.

**Results:**

528 responses were received. Education and advice on prescription and non-prescription medicines were the two top listed services provided. There were no significant differences in service provision between rural and metro based pharmacies. Many pharmacies were considering introducing new patient centred services. Four of the top ten frequently provided services have no public funding attached. Costs and staff availability are the most common barriers to undertake services, more predominantly in patient centred services.

**Conclusion:**

This study was the first to provide an evaluation of service provision in response to a new funding model for New Zealand Community Pharmacies. A broad range of services are being undertaken in New Zealand community pharmacies including patient-centred services. A number of barriers to service provision were identified. This study provides a baseline for the current levels of service provision upon which future studies can compare to and evaluate any changes in service provision with differing funding models going forward.

## Background

Due to pressures in funding and the desire to offer high quality health care services, there is an international drive by many governments to reform policy and redesign their health systems [1]. This comes in the face of aging populations and the burgeoning number of patients with multiple chronic conditions [2]. Across the global health sector two main mechanisms have underpinned this redesign. One is through “pay-for-performance” where payment is made for services and health targets achieved [2–4]. Another avenue has been to re-examine the traditional activities undertaken by various groupings of health professionals and to think how these roles could be extended to deliver services [5]. This “re-professionalisation” agenda is important for two professions, nursing and pharmacy [6–8] – particularly in light of the declining growth of primary care based medical doctors.

Traditionally pharmacists sourced ingredients, manufactured and supplied medicinal products. Over the past 50 years the pharmaceutical industry has taken over that role. [9] Internationally the re-professionalisation agenda for pharmacy has manifest through the implementation of policies that aim to expand the roles and refocus from that of dispenser and retailer to include more clinically-oriented patient-centred services that do social good. [10] Policymakers and pharmacy professional bodies have indicated through their vision, policy and action plan documents that they believe pharmacists should expand their roles and work ‘at the their top of their scope’ [11, 12].

Systematic reviews and meta-analyses suggest the provision of expanded clinical services have been successfully adopted and implemented by community pharmacists [13–15]. However, these programs have largely been stand-alone medicines management programs that pharmacists could opt into. Recently, New Zealand (NZ) has taken a system wide approach providing the biggest reform to New Zealand community pharmacy for 70 years. The new Community Pharmacy Services Agreement (CPSA), implemented in four stages between 2012 and 2014, has seen New Zealand community pharmacy change from a reimbursement per dispensing model to a patient-centred services model [16]. Like many counties, the strategy behind this new model is to create a sustainable pharmacy sector that is utilised for the extensive clinical skills and expertise of pharmacists. This is not an opt-in program and if pharmacists want to obtain funding through the CPSA then they need to participate in defined clinical programs. Pharmacies are a first point of contact for many patients entering the health system and the aim is to improve access and optimise medicines use through provision of high quality patient centred services that are integrated within primary care [17].

Much of the focus of the CPSA is shifting from volumes of medicines dispensed towards the safe and effective use of medicines in high needs patients through the provision of three main categories of services [17]. The funding model and service remuneration via the CPSA is complex; comparison to the funding structure in place in 2010 (pre-CPSA) is provided in Table 1. In addition to the call for improved service provision and health gain there was a fiscal drive for changing the funding mechanism. The previous “close control” mechanism allowed pharmacists in conjunction with the prescriber to increase the frequency of dispensing, for which a fee was payable to the pharmacy, where patients were deemed to require closer supervision of their medicines. This decision involved no risk sharing on the part of the pharmacy so almost inevitably grew exponentially, causing a significant fiscal overspend of national budget. The major change between these two agreements (the previous contract and the CPSA) is the introduction of the long term conditions service (LTC). This is a medicines management based service aimed at patients with multiple long term conditions and identified medicine adherence issues.

**Table 1:** Reimbursement of services pre-CPSA and with CPSA

Services	Description	Pharmacists fee for service pre CPSA (2010)	Pharmacists fee for service with CPSA (2015)
Core Services	Dispensing pharmaceuticals	$5.30 + mark-up (ranges from 4 to 5%)	$4.38 per initial item, $3 per repeat, multiplied by Relative Value Unit + 1 handling fee
Long term conditions (LTC) service	Medicines management service	–	as of 2015, $20 monthly fee per patient + above dispensing fees
Specific Services	e.g. methadone dispensing, aseptic Dispensing	2010 rates varied e.g. $5.30 (special foods)- $15.90 (aseptic services)	2015 rates vary e.g. $5.30 (for special foods)- $26.50 (for aseptic services)
DHB funded services (District Health Boards; responsible for the providing or funding the provision of health services in their district)	e.g. Medicine use reviews	Access to funding varies between DHBs	No CPSA funding. Access to funding varies between DHBs
Patient-funded services	e.g. emergency contraceptive pill	Patient pays full cost of consult and medicines	No CPSA funding. Patient pays full cost of consult and medicines

This new funding model is expected to generate incentives for pharmacies to provide for individual patient needs, rather than being financially rewarded for dispensing high volumes of pharmaceuticals. In essence pharmacies can still provide close supervision but share the financial risk associated with more frequent dispensing with the government for doing so. A focus on optimal use, medicines adherence and de-prescribing replaces the traditional model of pharmacists “churning out” as many prescriptions as they can in a day.

The Government (through the New Zealand Ministry of Health) has used the CPSA as a driver for change, riding on the notion that appropriate remuneration of service provision, is one of many factors that facilitates change in clinical practice [18, 19]. It is important to recognise that in this context (the addition of clinical services) structural and organisational changes are usually required to allow new practices to be incorporated into the routine workflow of community pharmacy [20]. This may include such things as consultation rooms and employing additional staff with the right expertise to undertake the required clinical activity called for by policy-makers. Consumers also need to be aware of these services and engage with their use, and there needs to be demand in-order for implementation to be successful. It has been demonstrated that if these factors are not adequately addressed they can become a barrier to undertaking or sustaining services; leading to financial difficulties for the pharmacy and sub-optimal care for patients [21]. It is unknown what the experience of the CPSA implementation is for New Zealand community pharmacy.

The April 2016 national reporting of the CPSA states that approximately 126,600 patients were registered with the LTC service across the country [22]. With current polypharmacy rates (approx. 35%) and adherence rates (ranging from 30 to 100%) it is expected that at least 500,000 New Zealanders would be eligible for the LTC service [23]. Year-on-year there has been an increase in dispensing of medicines and there was a decrease in the number of LTC patients enrolled in the program in April 2016 compared to April 2015. The year on year initial items growth is slightly above planning expectations [22]. It is difficult to interpret these high level data, although the current downward trend (since June 2014) in the number of LTC patients and the upward trend of total items dispensed (since June 2014) suggest that there has been limited success with the new funding model. Little is known about what specific services are being offered in New Zealand post the CPSA implementation and what influences service provision in the 958 community pharmacies contracted to provide services in New Zealand through the CPSA.

If community pharmacies are to provide more patient centred services is important to establish current levels of practice to provide a benchmark against which progress can be measured, and to identify enablers and barriers so that pharmacists can be supported to engage in this work. This information is also important for policy makers, pharmacy bodies and educators to plan the expansion of pharmacy services.

## Aims

Based on identified gaps, the aims of this study were to:

- Understand the demographic profile community pharmacies in New Zealand since the introduction of the CPSA;
- Understand the types of services offered in New Zealand community pharmacies since introduction of the CPSA;
- Investigate the number of patient centred services such as LTC are being offered by community pharmacies in New Zealand;
- Identify barriers to providing services related to the CPSA in New Zealand community pharmacies in the post CPSA environment
- Test a priori hypotheses about potential differences in service provision and barriers to service provision between pharmacies with respect to location (rural vs metropolitan) and type (banner vs independent pharmacies)

## Methods

Ethics approval for this project was obtained from the University of Otago Human Ethics Committee (Ref: D14/268).

### Research design

This study is largely quantitative with responses elicited through a national survey. Qualitative data was also collected through the option to free text answers for several questions in the survey.

### Sampling frame

A list of pharmacies in New Zealand was collated using publically available records, including the New Zealand Pharmacy Guild membership list (New Zealand Pharmacy Guild is a pharmacy owner’s organisation, membership is not compulsory), Google™ and the Yellow Pages Telephone Directory. A total sample of 1007 pharmacies was created from these sources. It was found that 49 pharmacies had closed, leaving a final survey sample of 958 pharmacies. Email addresses were available for 690 pharmacies representing close to three quarters (72%).

### Survey development

The survey instrument was developed through literature review and consideration of the research question and objectives. Developed by the authors over several months it consisted of the following sections; pharmacist and pharmacy demographic information [24], workforce and service provision, and barriers to undertaking services. To ensure face validity, the survey was piloted with 80 pharmacies from a geographically clustered sample, using electronic and postal methods [25]. A small number of changes were made to the structure and wording of the survey based on respondent’s feedback. Additional services were also included in the list of questions relating to service provision.

### Data collection

The survey was developed in SurveyMonkey® and the link was emailed in August 2014 to all community pharmacies with an email address (*n* = 690). All non-responders (*n* = 598) to the emailed survey and those without an email address (*n* = 268) were mailed a paper copy of the survey 2 weeks after the initial email, giving a total sample size of 958 pharmacies. A reminder email and postal drop was completed at both 5 and 7 weeks after the first email respectively. A third postal reminder was posted 11 weeks after the first email to all non-responders. A fourth and final email reminder was sent 5 months after the first email. The survey was promoted through the Pharmacy Guild of New Zealand newsletter (regular newsletter for pharmacy owners) and in Pharmacy Today - a sector-wide trade publication.

### Data analysis

Data entry was undertaken by a single individual (AS). If a survey had more than 20% of missing data, it was excluded from the data set. Data were analysed using SPSS (IBM analytics, version 23).

Demographic variables are presented as proportions of valid responses. Likert scale responses to opinions about barriers were coded as 1 = Strongly Agree (SA) to 5 = Strongly Disagree (SD). Responses were recoded into a 3-point ordinal variable (Strongly Agree (SA) + Agree (A), neutral, and Strongly Disagree (SD) + Disagree (D)) to compare agreement/disagreement while accounting for neutral responses. [26]. Comparisons between pharmacy location (rural vs metro) and types (banner vs non-banner) were undertaken using the chi-square test. [26] Statistical significance was assumed where *p* < 0.05. Services were ranked, with the service that is provided by the most pharmacies ranked as 1.

## Results

### Response rate

Five hundred and twenty eight surveys were returned, representing 55% of pharmacies in New Zealand (giving a response rate of 55%). Of these, 37% (*n* = 188) of surveys were completed online.

### Demographics of respondents

Nearly two thirds (*n* = 329, 62.3%) of respondents were pharmacy owners, had worked for > 20 years (*n* = 249, 47%) and worked between 41 and 50 h per week (*n* = 246, 47%). Over 40% of respondents were from major cities and just under this number identified as being located on a main street (39%). Interestingly, the large majority of respondents did not identify with belonging to a banner group (Independent pharmacies that are affiliated with a central office and pay fees for the right to use a recognized name and to participate in centralized buying, marketing, professional programs) (57%). There was a wide range (< 100–700+) in the number of prescriptions dispensed daily and over two-thirds (68%) of pharmacies supplied residential aged care facilities or community residential villages (Table 2).

**Table 2:** Demographic profile of community pharmacies

	**n (%)**
*Geographic Location*	
Major City	216 (41)
Provincial City	142 (27)
Provincial Town	136 (26)
Rural	26 (5)
*Physical Pharmacy Location*	
Main Street	206 (39)
By or within a medical centre	235 (44)
Within a suburban centre	111 (21)
In a mall	51 (10)
Supermarket	7 (1.3)
Internet pharmacy	1 (0.2)
*Number of days open*	
5 days	92 (17)
5.5 days	133 (25)
6 days	133 (15)
7 days	116 (22)
*Banner Group*	
No banner	302 (57)
Unichem/Unichem life	144 (27)
Vantage	52 (10)
Other banner	30 (6)
*Average number of prescriptions dispensed per day*	
< 100	54 (10)
101–200	162 (31)
201–300	133 (25)
301–500	116 (22)
501–700	28 (5)
> 700	12 (2)
*Top 5 languages spoken other than English*	
Chinese	207 (39)
Indian	61 (12)
Korean	51 (10)
Arabic	21 (4)
Maori	18 (3)
*Use of Social Media for advertising/pharmacy promotion*	
Facebook	213 (40)
Website	166 (31)
Smart phone application	16 (3)
Twitter account	6 (1)
*Training site services*	
Takes intern pharmacists	163 (31)
Takes undergraduate placement students	261 (49)
*Supply of medicines/services*	
RACF	164 (38)
Community residential village	129 (30)
Hospice	44 (10)
Prison	12 (3)
Private Hospital	31 (7)
Rural Depot Service	27 (6)
Public Hospital	26 (6)

*RACF* Residential aged care facility

Pharmacies were asked to estimate the proportion of time in a typical week that pharmacy staff spent on professional activities. Of the pharmacies who answered this question (*n* = 480), all except seven spent the majority of their week undertaking core services (dispensing medicines). On average, pharmacies spent 74% of their time spent on core services, 18.3% on LTC services, 6.3% on specific services, 3.8% on DHB funded services and 5% on non-funded services.

A large majority (96%) of pharmacies had patients enrolled in the LTC program, with the number of registrants ranging from 1 to 600.

Table 3 shows the number and proportion of pharmacies that undertake various services and the funding stream for the service. The service’s rank is also listed.

**Table 3:** Service provision by New Zealand community pharmacies

Funding Stream	Service	n (%)	Rank
Core funding	Prescription Dispensing	512 (97)	3
Specific Services funding	Extemporaneously Compounded Preparations Services	477 (90)	9
	Methadone Program	317 (60)	12
	Special Foods Services (e.g. gluten free)	316 (60)	13
	Clozapine Dispensing	272 (52)	16
	Needle Exchange	96 (18)	34
	Anticoagulation Services (e.g. CPAM)	96 (18)	35
	Aseptic Dispensing	37 (7)	38
	Syringe Driver Preparation	19 (4)	48
	Sterile Manufacturing services	3 (1)	54
DHB funding (Varies across DHBs)	Medicines Disposal	508 (96)	4
	Smoking Cessation Support Consultation Services	207 (39)	22
	Medicines Use Reviews	163 (31)	25
	Books on Prescription (self-help books for mental well-being)	24 (5)	45
	Medicine Therapy Assessment	24 (5)	46
LTC funded	Number with patients registered with LTC service (patients registered ranged from 1 to 600	508 (96)	4
Patient funded	ECP consultations	500 (95)	8
	UTI treatment (TMP accredited)	421 (80)	10
	Blood Pressure Measuring	268 (51)	17
	Sale of Continence Care Products	268 (51)	18
	Veterinary Dispensing	178 (34)	24
	Fitting Support Hosiery	154 (29)	27
	Equipment Hire	134 (25)	28
	Zinc testing	117 (22)	29
	Vaccinations (e.g. INTANZA)	117 (22)	30
	Blood Glucose	100 (19)	32
	Bone Density Scanning	48 (9)	36
	Bowel Health Screening e.g. faecal occult bloods	33 (6)	40
	Blood Cholesterol	14 (3)	49
	Iron testing	5 (1)	52
	Sleep Clinic	3 (1)	53
	Mole Mapping	2 (0)	55
No Funding	Education/Advice on prescription medicines to individuals	516 (98)	1
	Education/Advice on OTC medicines e.g. cold/flu	515 (98)	2
	Preparation and dispensing of Compliance aids (e.g. blister packs)	506 (96)	6
	Education/Advice on health concerns	505 (96)	7
	Home delivery service	415 (79)	11
	Education/Advice to community groups e.g. medicines taking issues, chronic disease	241 (46)	21
	Asthma Checks	42 (8)	37
	Visiting Allied Health Professionals e.g. midwife/child health nurse clinics	35 (7)	39
	Prescription depot service (e.g. supply rural site with no pharmacist)	31 (6)	41
	Internet Store	29 (6)	42
	24 Hour Service	25 (5)	43
	Automated Dispensing	25 (5)	44
	Drive through prescription drop-off	9 (2)	50
	Internet Dispensing	8 (2)	51
	Eyesight testing	1 (0)	56
Company sponsored	Hearing testing	20 (4)	47
	Weight Management Consultation Services	185 (35)	23

*ECP* emergency contraceptive pill, *CPAM* Community Pharmacy Anticoagulation Management service, *LTC* long term conditions, *TMP* trimethoprim accredited, *OTC* Over the counter pharmacy medicines (no prescription required)

### Pharmacy types and services

There were no significant differences in service provision between pharmacy location type except that rural pharmacies were more likely to provide prescription depot services (*p* < 0.01) and provincial towns were more likely to provide education and advice to community groups and needle exchange services compared to major and provincial cities (*p* < 0.01).

Banner pharmacies were significantly more likely (*p* < 0.01) to provide patient funded services compared to non-banner pharmacies, this included; bone density scanning, blood glucose, blood pressure measuring, bowel health screening, vaccinations, equipment hire, zinc testing, fitting support hosiery, selling continence care products and providing UTI treatment. There were no differences between banner/non-banner pharmacies or pharmacy location for the provision of core services, LTC services nor DHB funded services. Four of the top ten services ranked by pharmacists have no public funding attached, three of the four involving education and advice.

### Changes in service provision

One hundred and seventy four pharmacies (33%) stated they were ‘considering introducing a new service or screening/monitoring process’. The top five services that these pharmacies were considering were; Community Pharmacy Anticoagulation Management (*n* = 36), vaccinations (*n* = 35), blood glucose monitoring (*n* = 20), blood pressure monitoring (*n* = 18) and erectile dysfunction treatment (*n* = 17).

Eighty eight pharmacies (17%) had discontinued services, with blood cholesterol (*n* = 21) and zinc testing (*n* = 9) the most commonly stopped services due to them being too expensive.

### Barriers to undertaking services

**Fig. 1:**
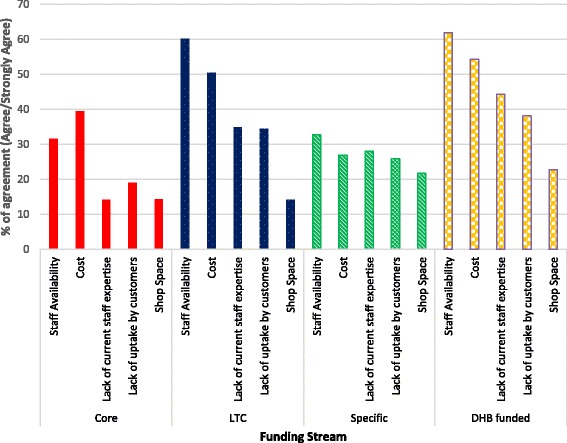
Barriers to undertaking community pharmacy services

Overall core service provision had the least barriers and DHB funded services the most. Across all funding streams, on average, staff availability was the most common barrier followed by cost to the pharmacy to undertake the services (Fig. 1). There were a number of free text comments around these, some examples are shown below;

‘*I would love to introduce new services. I find myself stretched very thin. Whilst the 'concept' of this new pharmacy services contract is great, execution is difficult. What I mean by that is there are so many elements to the work required that adding more services require more staff that require more $ to provide for. We are fortunate that our retail sales (esp. OTC) are strong. This in turn keeps the store going so that we can provide the few services that we do!’ (Participant 170)*

Many respondents commented about the extensive amount of time taken to complete an LTC patient review including the necessary paperwork and that the remuneration was not adequate.

*‘LTC is a waste of time because too much time spent form filling. The $20 payment only covers 8 minutes of a pharmacist’s time so not possible to do it in this time’ (Participant 40)*

*‘LTC , documentation -time taken to do this is biggest drawback, talking to customers is not an issue’ (Participant 49)*

### Pharmacy types and barriers

There were no differences in the barriers to providing services between pharmacy location (e.g. rural vs metro) except for rural pharmacies who were significantly more likely to report the lack of uptake of specific services by customers as a barrier compared with other location types (*p* = 0.03).

Banner pharmacies were significantly more likely to take undergraduate and intern students and dispense a high number of prescriptions a day (> 700), and be situated in a major city and a mall, than non-banner groups (*p* < 0.01). However there were no significant differences in the barriers to service provision between banner and non-banner pharmacies (*p* > 0.05).

## Discussion

There is limited evidence on how pharmacy is achieving against their new strategic goals while using this new funding model. Academic evaluation of the impact of recent New Zealand pharmacy policy change is limited to pharmacist perceptions of the CPSA agreement [27]. A short report indicates that many pharmacies are struggling to preform against the CPSA measurements [28]. It is expected that most community pharmacies will be providing the long term conditions (LTC) as this is required in-order to receive a CPSA contract. However, little is known about the services being provided by New Zealand community pharmacies under the current policy environment and the factors that are currently hindering and facilitating the implementation and ongoing sustainability of the sector. This study therefore aimed to provide the most comprehensive overview on community pharmacy and its service provision since the roll out of the CPSA model between 2012 and 2014.

### Community pharmacy in New Zealand

According to this study there is a range of pharmacy types in New Zealand but the most representative (labelled “typical”) is a non-banner pharmacy near a medical centre in a major city which is open for 5.5 days, has a pharmacist who speaks two languages, dispenses 101–200 prescriptions a day, uses Facebook, trains undergraduate students and provides services to residential aged care facilities. Despite the ‘city’ pharmacy being the most common, there is large variation in the types of pharmacies operating in New Zealand ranging from internet only pharmacies to rural and supermarket based pharmacies. The types of services offered were found to vary but only small dependence on the pharmacy type existed in this study. Interestingly a high proportion of respondents (approximately 70%) were from independent pharmacies.

Internationally, pharmacists are increasingly providing an extended range of accessible, high-quality, coordinated services that focus on patient care and population health and this study has found that New Zealand is no exception [29].

The vast majority of pharmacies are providing traditional pharmacy services; dispensing medicines and providing education/advice on medicines and health conditions, pharmacy staff also spend the majority of their day undertaking these services. There has been strong uptake of prescribing services (Emergency Contraceptive Pill (ECP) and trimethoprim provision for urinary tract infection (UTI)) but less on screening and health prevention activities at the time of this study. Whilst this aligns with the government’s goals of ‘care closer to home’ (improved access to medicines) it does not support the push to help people to make healthy choices and stay well, including; services for smoking cessation, weight management, sexual health and alcohol screening. A UK study of health professionals and consumers found less support for public health services undertaken through pharmacy compared to medicine services and minor ailments. This could also be true for the New Zealand setting, and therefore patient demand may be important in the implementation of these services [30–32].

Location had little effect on service provision with rural pharmacies providing similar services to that of their metro counterparts; however banner pharmacies were significantly more likely to provide patient-funded services than non-banner pharmacies. One study suggests that corporatisation (chain pharmacies) may influence the degree to which services are provided in a negative way but that was not seen in this study [33]. What is not known is whether the provision of these services are being driven by patient demand, as banner pharmacies are more likely to be situated in major cities or by the business model and/or the organisation structure and support from their banner group. If the later, it provides evidence that more support and assistance may be needed for independent pharmacies to enable equivalent extension of services and therefore increased patient access to services especially in rural areas.

Internationally there has been little work done on differences between independent and banner pharmacies. An Australian study found pharmacies with a high financial turn-over or younger owners were more likely to provide more enhanced pharmacy services [21]. Banner groups in Canada have higher turnover and lower running costs compared with independents [34].

There has been extensive uptake of the long-term conditions service with 95% of pharmacies having patients enrolled in the service. This is not unexpected as in-order to gain a dispensing contract, pharmacists need to provide LTC under the new CPSA funding arrangement. Unlike in other countries this is not an “opt-in” situation where the pharmacist decides whether or not to participate. There are limited quality assurance processes in place to ensure consistency across the service, pharmacists are required to complete a standardised registration form however it is then their choice how they record patient interactions in their Pharmacy Management Software [17]. Nationally there has been a lot of publicity and feedback from the sector around the inadequacy of the funding for this service and the extent of bureaucratic and cumbersome paperwork [16]. This study confirms media anecdote with staff availability and cost of providing the service being cited as significant barriers to carrying out these services by more than half of the respondents. This is consistent with findings from another recent NZ study where a perceived lack of remuneration and the required compliance around work pharmacists believe they were already doing, emerged as significant issues [27].

The staged rollout of the CPSA funding formula and the subsequent adjustments have attempted to alleviate these issues, however this study suggests that these issues are still seen by pharmacists as being the major barrier to optimal implementation of this service agreement. Remuneration for the provision of clinical services has been a long-time issue within the New Zealand community pharmacy sector and perceptions seem to have changed little within the sector over the past dozen years [27, 35, 36].

Whilst all but a few pharmacies have patients enrolled in the LTC service it is unknown if the patient-centric ethos is being upheld by all, especially those pharmacies who have enrolled in excess of 500 patients. It is also unknown if this model is providing different or better care and outcomes for patients than the previous volume based funding for dispensing. The present study did not aim to investigate this but it would be valuable to understand this further, this service is not offered in this way internationally therefore we are unable to learn lessons from other countries. To enable this to be investigated an LTC patient ‘flag’ within the administrative pharmacy claims database would help to evaluate this program by measuring hospitalisations and/or morbidity and mortality, through linked administrative databases.

There is a significant international literature reporting on the implementation of cognitive services but few studies which outline the broader pharmacy service provision following such a radical change in the funding model.

### Barriers to service provision

Core services had the least barriers for provision compared to all other types. On average, across the range of services provided, cost and staff availability are seen as the most significant barriers. This is consistent with international studies where it is known the presence of a remuneration scheme is not sufficient to guarantee uptake and sustainability of services of new cognitive services [10, 18, 19, 21, 27, 29, 35–40].

### Implications for policy and practice

This is the first NZ study to report pharmacy demographics and service provision following the introduction of the new community pharmacy funding model (CPSA). The findings of this study are useful in informing policy-makers and pharmacy professional organisations what is required to further advance the profession. This is especially the case with the new health strategy and pharmacy action plan being implemented by the Ministry of Health [11]. This study provides a baseline for the current levels of service provision upon which future studies can compare to and evaluate any changes in service provision with the CPSA going forward. From this study it seems that community pharmacists are providing a wide range of services. This study could be used by pharmacists to “speak up” take an advocacy platform and highlight not only the “what we do” but also the “how we do it” and the benefits to the community [41].

### Study limitations

A small number of pharmacies may have been missed in the sample due to the use of publicly available records which may not be up to date. However, our final sample corresponds very closely to that of Central Technical Advisory Services [22] and so we feel that our sample is largely representative. The response rate of 55% is comparable with another large NZ study [36] and although the findings of this study should not be generalised the response rate is acceptable within this field. Non-responder demographics are unknown and therefore the potential for non-responder bias warrants consideration.

### Implications for future research

This study provides a descriptive analysis of what services are being provided by community pharmacies, but not how well they are being provided. Further work is needed to understand how pharmacy is performing against other national health strategies e.g. becoming part of an integrated team to provide shared care or promoting optimal use of antimicrobial agents. The finding that there were no significant differences in service provision between pharmacy location and type is interesting. This breaks down stigma that mall pharmacies are “more retail” and observational research is required to explore this further. The finding that banner pharmacies provided a wider range of services than independent pharmacies warrants increased research around the corporatization of community pharmacy in New Zealand. There were no significant differences in the barriers to service provision between banner and non-banner pharmacies and so does this suggest that the sector is largely a homogeneous culture regardless of location or business model. More organisational culture studies are required to understand this further [42].

## Conclusion

This study set out to understand the types of services offered in New Zealand community pharmacies since introduction of a new funding model - the Community Pharmacy Services Agreement (CPSA). Determining the demographic organisational profiles of these pharmacies was also an aim. A national survey found that a broad range of services were being provided. There were no differences between banner/non-banner pharmacies or pharmacy location for the provision of core services, LTC services nor DHB funded services. Four of the top ten ranked services have no government funding attached, three of the four involving education and advice. Having adequate staffing to undertake extended service roles in addition to more traditional roles (i.e. dispensing) was the most common barrier to service provision followed by cost to the pharmacy of undertaking CPSA-related services.

## Abbreviations

CPSA: Community pharmacy services agreement
DHB: District health board
ECP: Emergency Contraceptive Pill
LTC: Long term conditions
NZ: New Zealand
OTC: Over the counter
UTI: Urinary tract infection
